# Can one teaspoon of trehalose a day mitigate metabolic syndrome and diabetes risks?

**DOI:** 10.1186/s12937-021-00685-6

**Published:** 2021-03-15

**Authors:** Fred Brouns, Ellen Blaak

**Affiliations:** Department of Human Biology, Faculty of Health, Medicine and Life Sciences, NUTRIM- School of Nutrition and Translational Research in Metabolism, Maastricht University, Maastricht, Netherlands, Post Box 616, MD 6200 Maastricht, Netherlands

Dear Madam/Sir

Trehalose is a disaccharide composed of 2 glucose units connected by an α-1,1 linkage, compared to maltose that has a similar disaccharide composition but differs in the saccharide bond (α-1,4). In humans, trehalose consumed at typical dietary rates is known to be digested in the small intestine by membrane bound trehalase, which splits the disaccharide into free glucose, which is available for subsequent absorption. The specific bond between the 2 glucose molecules of trehalose, however, impacts on its digestion rate, compared to maltose (a disaccharide of starch digestion), resulting in a slightly reduced absorption rate and consequently reduced glycemia, insulinemia and oxidation rate in energy metabolism, as we have shown in humans at rest and during exercise [1–4]. Trehalose is fully digestible, and glucose derived from trehalose is exactly the same molecule as glucose absorbed from other digestible carbohydrate sources such as starch. In this respect, the paper of Chiyo Yoshizane et al. [5] entitled “Daily consumption of one teaspoon of trehalose can help maintain glucose homeostasis: a double-blind, randomized controlled trial conducted in healthy volunteers”, raises a number of critical questions:

- Neither a clear hypothesis nor a discussion is presented about why free glucose, derived from trehalose, would lead to a different response compared to glucose absorbed from other carbohydrate sources.
- The authors failed to present a plausible mechanism explaining why glucose from trehalose is able to contribute to the prevention of pathologies that are predisposed to by postprandial hyperglycemia.
- The dose ingested, 3,3 g (13,2 kcal)/day, along with the normal daily food intake, is so small that a measurable effect on blood glucose, hormones, metabolic fate and energy deposition can neither be expected, nor can be measured. (In this respect, the authors may refer to an earlier paper in which they supplied 10 g/trehalose/day and compared it to sucrose, also claiming beneficial effects [6]. Yet, similar critics, see also next point, apply to that paper.
- Sucrose was given as placebo, which is different (half of it is fructose). We are concerned about this choice and ask why not maltose, a comparative disaccharide also composed to 2 glucose molecules, has been given, ruling out chances that any difference between TRE and SUC may be due to fructose rather than properties of trehalose.
- A potentially lower glycemic response, due to a lower digestion rate, is possible after ingestion of a larger dose of trehalose. That this would translate in a lower diabetes risk is hypothetical. A problem with the current study, in this respect, is that dose ingested is negligable.
- The study was financed and carried out by the company Hayashibara Co. Ltd, the producer of Trehalose, using its own employees as study participants, which causes the work to be subject to bias and to potentially driving for a favorable outcome.

Careful evaluation of the digestion, absorption and metabolism of trehalose substantiates that well controlled independent research is required before any conclusion can be drawn that the glucose absorbed from digested trehalose is able to impact on glucose homeostasis in favor of diabetes risk reduction. At present there are no data from well controlled independent studies that would allow to support the conclusions presented here by Chiyo Yoshizane et al. (2020).

## Data Availability

Not applicable.
